# Prevalence and severity of depressive symptoms in relation to rural-to-urban migration in India: a cross-sectional study

**DOI:** 10.1186/s40359-016-0152-1

**Published:** 2016-09-21

**Authors:** Hannah Maike Albers, Sanjay Kinra, K. V. Radha Krishna, Yoav Ben-Shlomo, Hannah Kuper

**Affiliations:** 1 Leibniz Institute for Prevention Research and Epidemiology—BIPS GmbH, Bremen, Germany; 2 Department of Non-Communicable Disease Epidemiology, London School of Hygiene & Tropical Medicine, London, UK; 3 National Institute of Nutrition, Hyderabad, India; 4 School of Social and Community Medicine, University of Bristol, Bristol, UK; 5 International Centre for Evidence in Disability, London School of Hygiene & Tropical Medicine, London, UK

**Keywords:** Rural-urban migrants, Depression, Mental health, Common mental disorders, Low and middle income countries

## Abstract

**Background:**

Migration is a major life event, which may also be a risk factor for depression. However, little is known regarding the relationship between these phenomena in low and middle income settings. This study explores the frequency and severity of depressive symptoms among rural-to-urban migrants compared to permanent rural and to urban residents in India.

**Methods:**

We assessed 884 subjects; urban non-migrants (*n* = 159), urban migrants (*n* = 461) and rural non-migrants (*n* = 264) in Hyderabad, India, in 2009–2010. The frequency and severity of depressive symptoms was assessed with the validated Telugu version of the Brief Patient Health Questionnaire. Multivariable logistic regression was used to examine the association between the presence of depressive symptoms and migration status while adjusting for gender, age and several sociodemographic and health-related parameters using Stata v.12.

**Results:**

The prevalence of mild to severe depressive symptoms was higher in women (11.3, 95 % confidence interval (CI) 8.3–14.3 %) compared to men (5.8 %, 95 % CI 3.7–7.9 %). Rural residents reported the highest prevalence of mild to severe depressive symptoms (women: 16.7 %, 95 % CI 9.8–23.5 %; men: 8.0 %, 95 % CI 3.7–12.3 %). Among women, the lowest prevalence was reported by migrants (8.2 %, 95 % CI 4.6–11.9 %). Among men, prevalence was similar in migrants (5.0 %, 95 % CI 2.2–7.7 %) and urban residents (3.9 %, 95 % CI 0–8.3 %). Multivariable logistic regression analyses showed no evidence for increased prevalence of mild to severe depressive symptoms among migrants compared to either rural or urban residents.

**Conclusions:**

There was no evidence for an increased prevalence of mild to severe depressive symptoms among rural-urban migrants compared to rural or urban residents.

## Background

Rural-to-urban migration occurs at high levels in India, a country with substantial rural-urban differences in economic development and job opportunities [1]. Migration is a major life event, which is associated with increased exposure to cardiovascular risk factors, and may also be a risk factor for depression [2, 3]. However, despite the increasing importance of rural-to-urban migration in low and middle income countries (LMIC), little is known regarding the relationship with depression. This is surprising since depression is highly prevalent globally and is the most common mental health disorder in primary care settings [4].

Migrants may be more vulnerable to depression as they are frequently exposed to stressors such as difficult environments in under-deprived urban areas, acculturation processes or discrimination experiences, loss of social support and family disruption [5–7]. On the other hand, those who migrate to urban settings may experience improvements in socioeconomic status (SES), living conditions and access to healthcare [5, 6], and therefore lower levels of depression compared to people who remain in rural areas. Furthermore, people who decide to migrate tend to be healthier and potentially less vulnerable to adverse health effects than those who remain in the rural population from which they originate; the so called “healthy migrant phenomenon” [8–10]. Urban non-migrants may therefore be expected to have the lowest prevalence of depression as they experience the beneficial aspects of the urban environment, without the potential stressor of migration.

In India, rural-urban migration is the fastest growing type of internal migration. There are about 100 million internal labour migrants (including also rural-rural migrants) who account for approx. 10 % of India’s Gross Domestic Product [11]. Rural-urban migrants commonly originate from poor economic backgrounds and marginalized population groups (i.e. Scheduled castes or Scheduled Tribes). They are frequently exposed to hazardous working and living conditions and face social and institutionalized discrimination. Nevertheless, rural-urban migration also represents an important pathway out of poverty [11].

Existing studies from LMICs report diverging results regarding the prevalence of depression among rural-to-urban migrants compared to rural or urban populations.

A number of studies have been conducted in China, reporting for the majority higher prevalence of depression or mood disorders among rural-urban migrants [12–19]). This is also true for a prospective study on forced resettlement [20]. However, the Chinese context differs from many other LMIC due to the ‘hukou system’ which restricts rural-urban migration and creates major barriers for migrants to access job opportunities, social services including health care or even schooling for their children in places other than their district of origin [14, 15].

Research from other LMIC is more scarce, but documents a similar trend. In two large longitudinal Indonesian studies migrants had the highest odds of ‘experiencing sadness’ compared to rural and urban non-migrants [5, 6]. A study conducted in Nepal found migrants to be particularly exposed to stress factors and suffering from more psychological distress while reporting less social support than non-migrants [21].

In contrast, results from South America are mixed. In a Peruvian study, common mental disorders were similarly prevalent among rural-urban migrants, rural and urban populations [22]. A study from Sao Paulo (Brazil) reported even lower odds of mood disorders among migrants compared to urban non-migrants, potentially as a result of the “healthy migrant phenomenon” [23].

We did not identify any study investigating mental health of rural-urban migrants in India. Closest in context are a limited number of studies among urban slum residents (of which many have rural origins) [24] or among internally displaced refugees [25]. These groups exhibit higher mental morbidity than the normal population. However, they are exposed to a variety of additional stressors, therefore their context is not comparable to the situation of voluntarily migrating labour workers.

There is thus a substantial knowledge gap regarding depressive disorders among the large and rapidly growing number of rural-urban migrants in India.

The aim of this study is to explore the prevalence of mild to severe depressive symptoms among rural-to-urban migrants compared to rural and also to urban non-migrants residing in and around Hyderabad, India. We also aim to describe the prevalence of individual depressive symptoms in each of the three migration groups. Based on the available information on living conditions of rural-urban migrants in India and in accordance with the majority of existing studies from other LMIC, we hypothesize that migrants experience more severe depressive symptoms than either rural or urban non-migrants.

## Methods

### Study population

The current analyses were based on cross-sectional data obtained during clinical investigations of participants from the Hyderabad arm of the Indian Migration Study (IMS), a previously studied cohort living in the city of Hyderabad, India, and its surrounding areas [2].

Participants were recruited via employer records of a large factory. All factory workers who self-reported to originate from rural areas and to reside in the city for at least 1 year were invited to participate, together with their co-migrant spouses (Rural-Urban Migrants). As rural comparison group, one sibling/close cousin per migrant and per spouse was invited, if still living in the village of origin (Rural residents). The urban comparison group consisted of a 25 % random sample of non-migrant factory workers, their urban spouses and siblings (Urban residents). The baseline was conducted between 2005 and 2007, during which time 1 995 participants were examined in Hyderabad (overall response rate for IMS 50 %).

All participants of the Hyderabad arm of the IMS were invited to a screening clinic at the National Institute of Nutrition between January 2009 and December 2010. The data collected during this follow-up phase were used for the presented analyses.

### Data collection

#### Questionnaire data

Participants were interviewed using a structured questionnaire. Migrant status was assessed based on self-reported migration history and current place of residence (rural/urban). The severity of depressive symptoms was assessed with the Brief Patient Health Questionnaire (BPHQ), a 9-item version of the Primary Care Evaluation of Mental Disorders questionnaire (PRIME-MD), which was designed to assist physicians in diagnosing depression [26, 27] and validated for use in Telugu [28].

Information on socio-demographic characteristics (age, gender, SES, marital status, children, household type, level of education, current occupation), alcohol and tobacco consumption were collected through interviewer-administered structured questionnaires. Physical activity (PA) over the past week was assessed based on the average amount of time and frequency of participation in different activities. SES was assessed with a sub-questionnaire of the Standard of Living Index (SLI) [29], a scale based on household assets.

#### Anthropometry

Weight was measured to the nearest 0.1 kg with digital Seca weighing machine (www.seca.com) and standing height to the nearest 1 mm with a plastic stadiometer (Leicester height measure; supplied by Chasmors, London). We used a validated oscillometric device (OMRON M5-; Omron, Matsusaka Co, Japan) to measure systolic blood pressure and diastolic blood pressure in the sitting position on the right arm using an appropriate sized cuff after a period of 5 min rest. We took three measurements, 2–3 min apart, and used the average of the last two measurements for analysis.

#### Bloods

Participants were asked to attend fasting and the time of the last meal was recorded. Venous blood samples (20 mL) were collected. IMS participants underwent a standard glucose tolerance test, unless they were diabetic or pregnant, and a second blood sample was taken after 2 h. Blood samples, with the exception of glucose assays, were separated and stored at -20 °C locally and transported to the Centre for Chronic Disease Control laboratory, New Delhi for insulin analysis. Insulin was assessed by ELISA method using kits from MERCODIA (Uppsala, Sweden). The quality of local assays was checked with regular external standards and internal duplicate assays and monitored by All India Institute of Medical Sciences (AIIMS). The Cardiac Biochemistry Lab, AIIMS, is part of the UK National External Quality Assessment (www.ukneqas.org.uk) programme and External Quality Assessment Scheme from RANDOX for quality assurance of Insulin and biochemical assays respectively. Glucose was measured on the day of the sample collection at the National Institute of Nutrition with the GOD-PAP method using RANDOX kits.

### Training and pilot testing

Training of fieldworkers and screening staff was conducted over a 2 week period and repeated at the mid-point of the study. A pilot study was conducted over a 1 week period.

### Statistical analyses

#### Exposure measure

The main exposure was a three-category variable distinguishing between rural-to-urban migrants, rural residents and urban residents. Participants were considered as rural-urban migrants if they originated from a rural area, had resided in an urban area for at least 1 year and were still living in the urban area (this also applies to those who migrated in-between baseline and follow-up study). Areas have been defined as rural or urban in accordance with the Indian census data based on municipal centers, population number, population density and proportion of the population engaged in agricultural production. Rural-urban migrants who returned to a rural place in-between studies were still considered as rural-urban migrants. Rural-urban migrants who had already returned prior to the baseline phase were classified as migrants if they had spent at least 50 % of their life in an urban area, otherwise they were considered as rural residents. Participants residing in an area reclassified from rural into urban between the baseline and follow-up studies were considered as rural residents. Individuals who originated from urban areas and had migrated to rural places were considered as urban residents if they had spent more than 90 % of their life in urban areas, otherwise they were excluded from analyses.

#### Outcome measures

Depressive symptoms were classified into ‘none/minimal’ (score 0–4) versus ‘mild to severe’ (score 5–27) [30]. The latter group also included participants on anti-depressant medication irrespective of current symptoms. Besides this severity rating, the BPHQ also allows categorical assessment of major depression and other depressive disorders [26, 30]. Classification of major depression requires the presence of at least five depressive symptoms over the past 2 weeks including one of the symptoms ‘depressed mood’ or ‘loss of interest’. ‘Other depressive disorders’ are sub-threshold disorders with two to four symptoms over 2 weeks, including ‘depressed mood’ or ‘loss of interest’.

#### Other measures

A diagnosis of diabetes was made using the World Health Organization fasting plasma glucose criterion of ≥7.0 mmol/l or 2 h post glucose load ≥11.1 mmol/l [31], or self report of a diagnosis of diabetes. Hypertension was defined through self report or blood pressure ≥140/90 mmHg. PA levels were calculated as daily hours of metabolic equivalents of task (MET), a combined measure reflecting total daily intensity and duration of a range of different activities.

#### Data analysis

All analyses were performed separately for men and women. This was based on the assumption that rural-urban migration may have a differential impact on men and women. In India, women traditionally leave their family and place of origin at the moment of marriage to live with their husband’s family [11]. The issue of family disruption due to rural-urban migration may thus be more challenging for men who would otherwise have stayed with their family of origin. On the other hand, men usually have the active role when deciding to migrate while women mainly accompany their husbands [11]. This may also impact on the way the migration process is perceived by both genders respectively and acts on their mental health.

Logistic regression with random effects was employed to account for sib-pair correlations when comparing rural-to-urban migrants and permanent rural and urban residents. Reported p-values were based on likelihood-ratio-tests. To avoid scarcity of data in subgroups (due to the low outcome prevalence, the three-categorical exposure variable, and gender-stratification of analyses), the main analyses were only adjusted for age. In exploratory analyses further adjustments were made for sociodemographic and health-related parameters. Due to missing covariate data, 4 persons were excluded from exploratory analyses (one man, three women). Analyses were performed with STATA v.12.

## Results

The response rate of the baseline study was 50 % [2]. Overall, 918 participants completed follow-up (46.0 % of baseline participants), of which 884 (44.3 % of baseline participants) were included in these analyses. Reason for excluding participants were incomplete BPHQ-data (*n* = 27) or unclear migration history (*n* = 7). The majority of migrants were permanent migrants (98 %). There were six return migrants (rural-urban-rural), two circular migrants (rural-urban-rural-urban) and two urban-rural migrants (the latter belong to those excluded from analyses).

Among included participants, 47.1 % (*n* = 416) were female (Table 1). There were 461 (52.1 %) migrants, 264 (29.9 %) rural and 159 (18.0 %) urban residents. The median time since migration was 29.4 years (interquartile range 23.4–35.2 years). The mean age of participants was 48.9 years (standard deviation 8.2 years, range 21.0–78.9 years). Male migrants were older than urban or rural residents, but there was no marked difference in age among the female migrant groups. Migrants had higher SLI than both urban and rural residents, among both males and females. They were less likely to live in extended families compared to rural residents. Among the males, migrants had higher levels of education than rural residents. Among females, migrants were less educated than urban residents and less likely to be employed compared to either urban or rural residents.

**Table 1:** Sociodemographic parameters by gender and exposure status (rural-Urban migrants, urban residents, rural residents)

Variable	Men (*N* = 468)			Women (*N* = 416)		
	Rural-urban migrants	Urban residents	Rural residents	Rural-urban migrants	Urban residents	Rural residents
Age (Years) Mean (Sd)	52.7 (5.9)	48.6 (9.0)	48.1 (10.2)	46.9 (6.0)	45.6 (7.2)	48.7 (10.3)
Standard Of Living Index Median (Iqr)	28 (26–30)	26 (22–29)	20 (15–23)	28 (26–30)	26 (24–30)	16 (13–20)
Education (N, %)						
No Formal Education	4 (1.7 %)	1 (1.3 %)	32 (21.3 %)	71 (32.4 %)	7 (8.4 %)	50 (43.9 %)
Up To Primary School	21 (8.7 %)	10 (13.2 %)	39 (26.0 %)	73 (33.3 %)	18 (21.7 %)	44 (38.6 %)
Secondary School	170 (70.3 %)	43 (56.6 %)	55 (36.7 %)	56 (25.6 %)	30 (36.1 %)	17 (14.9 %)
Professional Degree/Graduate/Post-Grad.	47 (19.4 %)	22 (29.0 %)	24 (16.0 %)	19 (8.7 %)	28 (33.7 %)	3 (2.6 %)
Household Type (N,%)						
Single/Shared Household Or Nuclear Family	170 (70.2 %)	54 (71.1 %)	95 (63.3 %)	158 (72.2 %)	63 (75.9 %)	66 (57.9 %)
Extended Family	72 (29.8 %)	22 (28.9 %)	55 (36.7 %)	61 (27.9 %)	20 (24.1 %)	48 (42.1 %)
Current Occupation (N, %)						
Not Employed	18 (7.4 %)	7 (9.2 %)	9 (6.0 %)	199 (90.9 %)	60 (72.3 %)	61 (53.5 %)
Manual	125 (51.7 %)	36 (47.4 %)	92 (61.3 %)	8 (3.7 %)	8 (9.6 %)	46 (40.4 %)
Non-Manual	99 (40.9 %)	33 (43.4 %)	49 (32.7 %)	12 (5.5 %)	15 (18.1 %)	7 (6.1 %)
Marital Status (N, %)						
Currently Married	238 (98.4 %)	70 (92.1 %)	148 (98.7 %)	209 (95.4 %)	78 (94.0 %)	80 (70.2 %)
Not Married	4 (1.7 %)	6 (7.9 %)	2 (1.3 %)	10 (4.6 %)	5 (6.0 %)	34 (29.8 %)
No. Of Children (N, %)						
0–2	99 (42.9 %)	38 (55.9 %)	71 (52.2 %)	89 (43.2 %)	44 (56.4 %)	47 (44.3 %)
3	84 (36.4 %)	19 (27.9 %)	33 (24.3 %)	77 (37.4 %)	24 (30.8 %)	34 (32.1 %)
≥ 4	48 (20.8 %)	11 (16.2 %)	32 (23.5 %)	40 (19.4 %)	10 (12.8 %)	25 (23.6 %)

* Age adjusted *P*-value < 0.05 in comparison to rural-urban-migrants

** Age adjusted *P*-value < 0.01 In comparison to rural-urban-migrants

The prevalence of any depressive disorder (major depression/other depressive disorder) was 3.1 % in women (95 % confidence interval (CI) 1.5–4.8 %) and 3.2 % (1.6–4.8 %) in men. Due to the low outcome prevalence, subsequent results refer to the severity of depressive symptoms (‘none/minimal’ versus ‘mild to severe’) rather than categorical diagnoses (Tables 2 and 3). The prevalence of mild to severe depressive symptoms was higher in women (11.3 %, 95 % CI 8.3–14.3 %) compared to men (5.8 %, 95 % CI 3.7–7.9 %). Among men, rural residents had the highest prevalence of mild to severe symptoms (8.0 %, 95 % CI 3.7–12.3 %) while the prevalence was similar in migrants (5.0 %, 95 % CI 2.2–7.7 %) and urban residents (3.9 %, 95 % CI 0–8.3 %). The prevalence of each individual symptom was consistently lowest in migrants and highest in rural residents. Among women, the prevalence of mild to severe symptoms was highest among rural residents (16.7 %, 95 % CI 9.8–23.5 %) and lowest among migrants (8.2 %, 95 % CI 4.6–11.9 %).

**Table 2:** Frequency and distribution of depressive symptoms by exposure status among women

Variable	Women				
	Total (N)	Rural-urban migrants (N,%)	Urban residents (N,%)	Rural residents (N,%)	*P*-value ^a^
Presence Of Individual Symptoms: ^b^					
Decreased Interest Or Pleasure	90	47 (21.5)	16 (19.3)	27 (23.7)	0.88
Depressed Mood	68	29 (13.2)	15 (18.1)	24 (21.1)	0.06
Insomnia Or Hypersomnia	94	46 (21.0)	21 (25.3)	27 (23.7)	0.82
Fatigue Or Loss Of Energy	96	47 (21.5)	17 (20.5)	32 (28.1)	0.60
Poor Appetite Or Overeating	43	17 (7.8)	11 (13.3)	15 (13.2)	0.30
Feelings Of Worthlessness Or Inappropriate Guilt	20	7 (3.2)	7 (8.4)	6 (5.3)	0.18
Diminished Ability To Think Or Concentrate	21	9 (4.1)	2 (2.4)	10 (8.8)	0.41
Psychomotor Retardation Or Agitation	42	19 (8.7)	7 (8.4)	16 (14.0)	0.67
Thoughts Of Death Or Suicide Or Self-Harm	20	6 (2.7)	4 (4.8)	10 (8.8)	0.07
Overall Severity Of Symptoms:					
None/Minimal Symptoms	369	201 (91.8)	73 (87.9)	95 (83.3)	0.14
Mild To Severe Symptoms ^c^	47	18 (8.2)	10 (12.1)	19 (16.7)	
Presence Of Any Depressive Disorder ^d^	13	3 (1.4)	7 (8.4)	3 (2.6)	Na ^e^
Total	416	219	83	114	

^a^ Evidence for a difference across exposure groups; *P*-values derived from likelihood ratio tests based on logistic regression with random effects to account for sib-pair correlations; age-adjusted

^b^ According to the patient health questionnaire

^c^ Including 2 women without current symptoms but on anti-depressant medication

^d^ Including major depression and other depressive disorders

^e^ Not applicable (scarcity of data)

**Table 3:** Frequency and distribution of depressive symptoms by exposure status among men

Variable	Men				
	Total (N)	Rural-urban migrants (N,%)	Urban residents (N,%)	Rural residents (N,%)	*P*-value ^a^
Presence Of Individual Symptoms: ^b^					
Decreased Interest Or Pleasure	66	23 (9.5)	8 (10.5)	35 (23.3)	0.001
Depressed Mood	35	14 (5.8)	6 (7.9)	15 (10.0)	0.41
Insomnia Or Hypersomnia	44	18 (7.4)	8 (10.5)	18 (12.0)	0.22
Fatigue Or Loss Of Energy	42	15 (6.2)	9 (11.8)	18 (12.0)	0.05
Poor Appetite Or Overeating	37	11 (4.6)	4 (5.3)	22 (14.7)	0.004
Feelings Of Worthlessness Or Inappropriate Guilt	15	4 (1.7)	3 (3.9)	8 (5.3)	0.46
Diminished Ability To Think Or Concentrate	14	5 (2.1)	2 (2.6)	7 (4.7)	0.30
Psychomotor Retardation Or Agitation	17	6 (2.5)	3 (4.0)	8 (5.3)	0.16
Thoughts Of Death Or Suicide Or Self-Harm	10	2 (0.8)	2 (2.6)	6 (4.0)	0.06
Overall Severity Of Symptoms:					
None/Minimal Symptoms	441	230 (95.0)	73 (96.1)	138 (92.0)	0.24
Mild To Severe Symptoms ^c^	27	12 (5.0)	3 (3.9)	12 (8.0)	
Presence Of Any Depressive Disorder ^d^	15	6 (2.5)	1 (1.3)	8 (5.3)	Na ^e^
Total	468	242	76	150	

^a^ Evidence for a difference across exposure groups; *P*-values derived from likelihood ratio tests based on logistic regression with random effects to account for sib-pair correlations; age-adjusted

^b^ according to the patient health questionnaire

^c^ Including 3 men without current symptoms but on anti-depressant medication

^d^ Including major depression and other depressive disorders

^e^ Not applicable (scarcity of data)

Among women, both urban residents (age-adjusted OR 1.6, 95 % CI 0.5–4.9) and rural residents (age-adjusted OR 2.5, 95 % CI 1.0–6.5) appeared to have more depressive symptoms than rural-urban migrants, but this did not reach statistical significance (Table 4). These associations changed little after adjustment for socioeconomic and health parameters.

**Table 4:** Odds ratios (Ors) for the presence of mild to severe depressive symptoms across exposure groups

	Total	Presence of mild to severe depressive symptoms	Model I: age-adjusted	Model Ii: adjusted for socioeconomic parameters ^a^	Model Iii: adjusted for socioeconomic and health parameters ^b^
	N	N (%)	Or (95 % Ci)	Or (95 % Ci)	Or (95 % Ci)
Women			(*N* = 416)	(*N* = 413)	(*N* = 413)
Rural-To-Urban Migrants	219	18 (8.2 %)	Baseline	Baseline	Baseline
Urban Residents	83	10 (12.1 %)	1.6 (0.5–4.9)	1.9 (0.6–5.6)	1.7 (0.6–5.2)
Rural Residents	114	19 (16.7 %)	2.5 (1.0–6.5)	2.9 (0.8–10.5)	2.9 (0.8–10.8)
Men			(*N* = 468)	(*N* = 467)	(*N* = 467)
Rural-To-Urban Migrants	242	12 (5.0 %)	Baseline	Baseline	Baseline
Urban Residents	76	3 (4.0 %)	1.0 (0.2–5.0)	1.1 (0.2–5.5)	0.8 (0.1–5.7)
Rural Residents	150	12 (8.0 %)	2.4 (0.8–7.4)	2.3 (0.5–11.0)	2.2 (0.3–15.3)

^a^ Adjusted for age, socioeconomic status, education, household type (extended family vs. single/shared household/nuclear family)

^b^ Adjusted for age, socioeconomic status, education, household type, physical activity (metabolic equivalents of task, mets), body mass index, hypertension, diabetes

Among men, there was no difference in depressive symptoms between rural-urban migrants and urban residents (age-adjusted OR 1.0, 95 % CI 0.2–5.0). In contrast, rural residents appeared more likely to have depressive symptoms than rural-urban migrants, although this did not reach statistical significance (age-adjusted OR 2.4, 95 % CI 0.8–7.4). Again, adjustment for socioeconomic and health parameters did little to change these associations.

## Discussion

In this study, the prevalence of any depressive disorder (major depression/other depressive disorder) was approximately 3 %. This is consistent with the majority of earlier studies among India’s general population [7, 32, 33] although it has to be noted that these have been criticized to potentially underestimate the true prevalence [34]. The similar depression prevalence in men and women in this study conflicts with the usually observed higher prevalence in women [4, 35, 36]. We did however observe a higher prevalence of mild to severe depressive symptoms among women.

Contrary to our hypothesis, there was no evidence for an increased severity of depressive symptoms among migrants compared to either rural or urban residents. Instead, our study revealed a consistent trend of higher prevalence of depressive symptoms among rural residents compared to migrants or urban residents, though this was not statistically significant. This contrasts with most earlier studies in other LMIC which reported higher mental morbidity among rural-urban migrants compared to rural and urban populations [13–19]. There are several potential explanations for this discrepancy. First, the study may have been under-powered and consequently this may be a chance finding. Second, any differences may have been the result of uncontrolled confounding, given that the associations were attenuated after multivariable adjustment. We can also suggest several possible explanations for why the prevalence of depressive symptoms could be higher among rural non-migrants, than migrants. Lower depression levels among migrants compared to rural residents might have been caused by self-selection effects with healthier people being more likely to migrate (healthy-migrant-phenomenon).

Alternatively, the lower prevalence of symptoms among migrants could be related to an improvement in living conditions and reduction in risk factors when moving from rural to urban environments (e.g. better job opportunities).

Our study included only employed migrants with relatively long duration of migration. This is in line with previous research showing that secure working positions are associated with better mental health among rural-urban migrants [37, 38]. Two studies which also included only employed migrants reported a similar trend as observed by us (i.e. good mental health among rural-urban migrants) [12, 39].

Regarding the duration of migration, there is evidence of an association between shorter duration of stay and more discriminatory experiences among rural-urban migrants [40]. Furthermore, acculturative stress and discriminatory experiences have been identified as important correlates of depression among internal migrants [19, 38, 41]. We hypothesize that successful sociocultural adaptation after several years of urban residence may provide a further explanation for the similarity in depression levels between migrants and the urban population in our study.

In consequence, this would indicate that rural-urban migration might reduce depression levels under certain conditions (e.g. successful adaptation, secure working position). This reflection is of course purely hypothetical and needs further verification by longitudinal studies.

### Strengths and limitations

Our study is the first to investigate the relationship between rural-urban migration and depression in India, one of the largest LMIC characterized by substantial inflows of rural migrants into urban areas. It overcomes limitations of several earlier studies in LMIC by including an urban as well as a rural comparison group [16, 23, 42]. The sib-pair design is unique in this area of research [43] and allows to control for some unknown or unmeasured confounders (e.g. genetic or childhood parameters) when comparing rural-urban-migrants to their sibs, as well as for secular trends which may have been simultaneously experienced by migrants and their sibs. The outcome was measured with a standardised tool used frequently in India and other LMICs [28, 44–47], which had been validated in the local language [28]. Furthermore, there was almost no missing data.

Limitations of our study include the following: The current analyses were conducted within the context of a cohort study, and so the sample size constrained to the originally recruited subjects. In addition, the low response rate further reduced the sample size. As a consequence, there was limited power for the study to detect small difference in prevalence of depressive symptoms. The low response rate may also have introduced selection bias. There was no evidence for age or gender differences between participants who completed follow-up or were lost to follow-up. However, our data were insufficient to further explore differential participation by individual characteristics or to use statistical measures to adjust for the low response rate. Non-response is a common problem in migrant studies, partially due to the inherent mobility of migrant populations. Furthermore, we lacked depression assessment pre-migration. Due to limited sample size, analyses were only controlled for age and gender, residual confounding may thus be present. Regarding exposure measurement, some geographical areas were reclassified from rural to urban over time (based on Indian census data), and the mental health implications are unclear for people living in these areas, given the lack of available data. Finally, the duration of migration may play an important role in the expression of depressive symptoms, e.g. regarding adaptation processes. Unfortunately, we did not include adaptation measures in our study. Since the sample included mainly permanent labour migrants, results should only be generalized to this subgroup.

Within this study a symptom-based definition was chosen as the primary outcome (i.e. presence of depressive symptoms) rather than the prevalence of depression itself. Symptom presentation of depression may vary cross-culturally [48, 49], raising concerns about the validity of international classification systems [50, 51]. Initially, it was intended to use depression score as continuous measure to maximise the use of available information. However, due to its highly skewed distribution and large number of zero-values, the required normal transformation was not feasible. A low cut-off value was then used to define the presence of depressive symptoms, due to concerns about potential underreporting of symptoms in the Indian context (e.g. due to fear of stigmatisation) [34, 52] and to maximise the power available. We have repeated the modeling analyses using two different cut-offs (8 and 10 points) to define the presence of depressive symptoms. The results are very similar except for much wider confidence intervals and very small numbers in some subgroups (available on request).

## Conclusions

In conclusion‚ among long-term rural-to-urban migrants there is no evidence of increased depressive symptoms compared to rural or urban residents. Our results underline the fact that rural-urban migrants are a heterogeneous population, which should be taken into account when designing and interpreting migrant studies. Longitudinal studies incorporating recent and longer-term migrants with repeated measures of depressive symptoms and adaptive behaviours would be useful in understanding psychopathology associated with rural-urban migration.

## Abbreviations

BPHQ: Brief Patient Health Questionnaire
CI: Confidence interval
IMS: Indian Migration Study
LMIC: Low and middle income countries
MET: Metabolic equivalents of task
OR: Odds Ratio
PA: Physical activity
PRIME-MD: Primary Care Evaluation of Mental Disorders Questionnaire
SES: Socioeconomic status
SLI: Standard of Living Index
